# Factors associated with cervical cancer screening: results from cross-sectional surveys in Kenya and Malawi

**DOI:** 10.1186/s12889-025-23143-y

**Published:** 2025-05-27

**Authors:** Corrina Moucheraud, Symon Chibaka, Ginger Golub, Pericles Kalande, Amos Makwaya, Eric Ochieng, Vitalis Ogutu, Khumbo Phiri, Sam Phiri, Risa M. Hoffman

**Affiliations:** 1 School of Global Public Health, New York University, New York, USA; 2 Children in the Wilderness, Lilongwe, Malawi; 3 Innovations for Poverty Action, Nairobi, Kenya; 4 Partners in Hope, Lilongwe, Malawi; 5 David Geffen School of Medicine, Department of Medicine, Division of Infectious Diseases, University of California, Los Angeles, USA

**Keywords:** Cervical cancer screening, Kenya, Malawi

## Abstract

**Background:**

Cervical cancer screening is an essential public health intervention, and critical to meeting the Global Strategy for Cervical Cancer Elimination goals – yet most women in low- and middle-income countries are never screened. There is a need to understand context-specific factors that facilitate or prevent women from engaging in screening.

**Methods:**

This analysis leverages data collected in 2022–2023 from a national mobile phone-based survey in Kenya and from a household survey conducted in three districts of Malawi. Informed by the Health Belief Model, we assess whether women’s reported cervical cancer screening history (ever or never screened) was associated with their perceived susceptibility (awareness of cervical cancer risk factors), perceived severity (knowing someone who was affected by cervical cancer), perceived barriers (access to services), perceived benefits (trust in information about cervical cancer prevention), self-efficacy (engagement in other preventive health behaviors), and cues to action (speaking with others about cervical cancer prevention).

**Results:**

Ever-screening for cervical cancer was reported by 49.7% of the 736 Kenyan respondents and 42.5% of the 261 Malawian respondents. There were few associations between women’s demographic or socioeconomic characteristics and screening history. The strongest associations were seen for cues to action (women who had spoken about cervical cancer with health workers had 1.88 the adjusted risk ratio for screening in Kenya [95% CI 1.59, 2.24] and 1.89 the adjusted risk in Malawi [95% CI 1.41, 2.54] compared to women who never had these conversations); and for knowing someone who had, or who had died due to, cervical cancer (aRR 1.34 and 1.30 respectively in Kenya, and aRR 2.03 and 1.46 respectively in Malawi). In both countries, self-efficacy was also associated with screening, as was perceived severity in both countries (i.e., knowing someone who had, or who had died due to cervical cancer, which was reported by many Kenyan and Malawian respondents). In Kenya, knowledge of cervical cancer risk factors was also associated with women’s screening history, as was access to other preventive health services in Malawi.

**Conclusions:**

These results suggest promising areas for interventions aiming to increase cervical cancer screening in these contexts: encouraging health workers to discuss screening with eligible women, leveraging women’s peers who have been affected by cervical cancer, and promoting screening during other preventive health services.

**Supplementary Information:**

The online version contains supplementary material available at 10.1186/s12889-025-23143-y.

## Background

Cervical cancer is a major global public health challenge and inequity [1]. Globally, there are approximately 660,000 new cases of cervical cancer each year and 348,000 deaths, and more than 90% of these cases and deaths occur in low- and middle-income countries (LMICs) [2]. While cervical cancer incidence is declining in many countries [3] and cervical cancer is one of the only cancers with demonstrated improvements in premature mortality globally [4], these gains are largely concentrated in high-income countries, where women have better access to highly effective primary and secondary prevention strategies [2, 5, 6], Women in LMICs are therefore not seeing corresponding improvements in cervical cancer outcomes [3, 7, 8]. These access barriers to primary and secondary prevention strategies in LMICs are due to myriad factors from individual to societal levels [9, 10].

In 2020, the World Health Assembly adopted the Global Strategy for Cervical Cancer Elimination, whereby each country should achieve 90% coverage of primary prevention (girls fully vaccinated against human papillomavirus (HPV) before age 15), 70% coverage of secondary prevention, and 90% of tertiary prevention by the year 2030 [11]. If countries meet these targets, the world would see 74 million fewer cases of cervical cancer and 62 million fewer deaths attributable to cervical cancer over the next century [11]. In LMICs alone, meeting these targets would cause a one-third reduction in premature mortality related to cervical cancer over the next decade [12]. Modeled analyses have demonstrated that widespread screening is essential for achieving these health impacts (i.e., HPV vaccination alone is not sufficient) [12, 13].

Despite the importance of cervical cancer screening for individual- and population-level health outcomes, most women in LMICs have never been screened. Three recent analyses have estimated the pooled lifetime ever-screened prevalence among women in LMICs as 27% [14] and 43.6% [15], and 10.5% among women in Africa [16]. Many studies have identified important intra-national differences in uptake of cervical cancer screening, with associated factors such as social and demographic characteristics (wealth, educational attainment, etc.) [16–19], and attitudes about cervical cancer and about cervical cancer screening [18–21].

It is essential to understand who is being screened and who is not, in order to design effective interventions to reach unscreened women. As influences on screening behavior may vary across contexts and over time, this study aims to provide information from two high-burden countries in Eastern and Southern Africa. The objective of this paper is to identify potentially intervention-amenable factors to improve screening for Kenyan and Malawian women. These countries were selected because they are geographically proximate in Eastern Africa, their cervical cancer screening guidelines are similar, and they experience high cervical cancer burden. We explore this question in each country using the same framework and constructs, and present findings separately for each country due to differences in sampling and reflect on overall differences and similarities. We aim to fill a gap in the literature with a theory-informed, community-based, quantitative exploration of this important public health topic in two high-burden countries.

## Methods

This analysis is nested within a larger study that surveyed parents of preadolescent girls about their experiences with HPV vaccination; as this study population includes women eligible for cervical cancer screening, the survey also asked about women’s histories of cervical cancer screening.

Conceptual model: This study uses the Health Belief Model (HBM) to explore factors hypothesized to be associated with whether a woman has been screened for cervical cancer. The HBM was developed to leverage health psychology and behavioral theories to posit that a person’s health behavior is affected by their perceptions about the health issue (its threat, i.e., seriousness and severity) and of the health behavior itself (its benefits and costs), with potential impact of demographic characteristics, and possibly influenced by a person’s self-efficacy and their exposure to cues to act [22].

Table 1 shows how our study operationalized the HBM constructs to study factors associated with cervical cancer screening.

**Table 1:** Health Belief Model constructs and how operationalized and measured in this study

HBM construct	Operationalization in this study	Measurement in this study
Perceived susceptibility	Knowledge about the cause of cervical cancer	4 survey questions, each dichotomized (correct versus incorrect or don’t know); and a summative score of correct responses
Perceived severity	First-hand familiarity with cervical cancer	One multi-select survey question about knowing someone who had and survived/or who had died from cervical cancer, each dichotomized (knew someone versus did not know anyone)
Perceived barriers	Reported access to preventive health care services	Two survey questions about difficulty in accessing services (routine vaccination, and HPV vaccination for one’s children), each on a 1-4 scale (not at all difficult, to very difficult)
Perceived benefits	Reported trust in official information about cervical cancer prevention	Two survey questions about trusting information about HPV vaccination (from the government/Ministry of Health and from health workers) each dichotomized (a lot or some, versus not much or not at all or [refer not to answer)
Self-efficacy	Participation in other preventive health behaviors	One survey question about having received any doses of a COVID-19 vaccine, dichotomized (any doses, versus no doses)
Cues to action	Having spoken about cervical cancer or cervical cancer prevention	Two survey questions about ever speaking about cervical cancer and or HPV vaccination (with a health worker, or with other parents), each dichotomized (ever versus never)

### Survey instrument

We developed a survey that included questions as indicated in Table 1; these constructs have not specifically been validated for applicability of the HBM and cervical cancer screening in these contexts. Perceived susceptibility was measured via knowledge about cervical cancer, and this was assessed through four questions that our team has used previously in these settings [23, 24]. Perceived severity was assessed by asking whether the respondent knew someone who had, or had died from, cervical cancer. Perceived barriers were proxied by access to another important routine preventive health behavior—vaccination—and measured using items from the World Health Organization Behavioural and Social Drivers of Vaccination tools [25], as was “cues to action.” Lastly, self-efficacy was represented by whether the person had received any doses of the COVID-19 vaccine, as a way to assess their engagement in other preventive health care. Female respondents were also asked “Have you ever been screened/tested for cervical cancer?” (response options were Yes or No). In addition, we asked about the respondent’s sociodemographic characteristics. We also included a survey module to measure social desirability bias, per the five-item Socially Desirable Response Set (SDRS-5) [26]. The same questions were asked in both Kenya and Malawi. The survey instrument was developed in English, translated into local languages for the study countries, and formatted for interviewer-led data collection using tablet-based survey software (SurveyCTO). The survey questions used in this analysis can be found in the Supplemental file.

### Study settings

Kenya has the 26th-highest age-standardized incidence of cervical cancer globally (32.8 cases per 100,000 women) and the 24th-highest age-standardized attributable mortality (21.4 deaths per 100,000 women) [2]. Malawi has the third-highest age-standardized cervical cancer incidence (70.9 cases per 100,000 women) and second-highest age-standardized attributable mortality (54.1 deaths per 100,000 women) globally [2]. In both Kenya and Malawi, national policies (the Kenya National Cancer Screening Guidelines [27] and the Malawi National Service Delivery Guidelines for Cervical Cancer Prevention and Control [28]) endorse routine cervical cancer screening for women aged 25–49 years; depending on which screening test is used and characteristics of the woman (e.g., HIV status or previous test results), the recommended screening interval varies. According to recent household surveys, approximately 17% of women in each country have ever been screened for cervical cancer [29, 30]. Previous studies from Kenya and Malawi have found that cervical cancer screening is associated with women’s sociodemographic characteristics (especially greater educational attainment) [31–33], awareness and knowledge about cervical cancer and screening [34–37], and health system characteristics (e.g., access to a health facility, quantity and quality of the health workforce, and availability of screening supplies) [34, 38–41].

### Data collection

In Kenya, we conducted a random-digit dialed phone survey; in Malawi, we conducted a household survey. A phone survey was conducted in Kenya due to very high mobile phone penetration (the most recent Afrobarometer survey found that over 93% of Kenyan women and over 95% of Kenyan men own a mobile phone [42]) while in Malawi, where mobile phones are not as prevalent, a household survey was used. In both countries, eligible respondents were parents or caregivers of preadolescent girls (those currently or recently age-eligible for HPV vaccination), as this was the focus of the parent study Further details on recruitment and sample selection can be found in publications from the parent studies [23, 24]. Data were collected between July and October 2022 in Kenya, and between December 2022 and January 2023 in Malawi. The sample size for the survey in each country was determined for the parent study (about HPV vaccination) based on estimated HPV vaccine coverage; and the response rate to the phone survey in Kenya was 97.4% among those eligible, and in Malawi, 90.6% of eligible households visited completed the survey.

### Data analysis

This analysis uses data from female survey respondents only given its focus on self-reported cervical cancer screening. As the associations between HBM constructs and cervical cancer screening behavior have been found to vary across countries [20], and as the samples differ due to different data collection and sampling procedures in each country, we analyzed the survey data from Kenya and Malawi in identical but separate models. We generated descriptive statistics for the study population, and for the dependent variable (reported having ever been screened for cervical cancer) and independent variables (per the HBM, as shown in Table 1). We used GLM Poisson regression models with robust error variance [43] to explore associations between the independent and dependent variables, including covariates to capture characteristics of the respondent: age, employment status, marital status, educational attainment, household income sufficiency, area of residence, household size, and social desirability score.

### Ethical review

The Kenya survey was reviewed and approved by the University of California Los Angeles Institutional Review Board (#22–000005) and by the Kenya Medical Research Institute Scientific and Ethics Review Unit (#SERU4456), and was granted a research permit by the Kenya National Commission for Science, Technology and Innovation (#821,192). The Malawi survey was reviewed and approved by the Malawi National Health Sciences Research Committee (protocol #21/04/2685) and by the University of California Los Angeles Institutional Review Board (protocol #21–001174). All respondents gave oral informed consent to participate, and the research was conducted in accordance with the Declaration of Helsinki.

## Results

In total, 736 Kenyan women and 261 Malawian women contributed data to this analysis. Characteristics of the sample are shown in Table 2. Having ever been screened was reported by 49.7% of surveyed women in Kenya (*n* = 366) and 42.5% of surveyed women in Malawi (*n* = 111). The average age of surveyed women was 37.9 years in Kenya and 39.1 years in Malawi, and screening status was significantly associated with older age in Kenya (but not in Malawi): the average age of screened Kenyan women was 39.8 years and of unscreened women was 35.9 years, while in Malawi the average age of screened women was 38.7 years and of unscreened women was 39.4 years. Kenyan women who were employed more commonly had been screened than their unemployed counterparts (52.3% versus 42.5%, respectively); this association was not seen in Malawi. Most women in both countries were married (67.0% in Kenya and 74.0% in Malawi), and married women in Malawi were slightly more commonly screened than their unmarried peers, although this was not statistically significant. The distribution of educational attainment was very different in the two countries: 69.4% of surveyed Malawian women reported less than a primary level education, whereas 66.2% of women in Kenya reported having completed secondary school or an advanced degree. Household income sufficiency was worse in Kenya, with 56.9% of women reporting that it was insufficient versus only 24.9% of women in Malawi reporting this. The social desirability score was slightly higher on average in Malawi (3.2 on a scale of 0 to 5) than in Kenya (2.1 on a scale of 0 to 5).

**Table 2:** Characteristics of women in the sample, overall and by cervical cancer screening behavior

	**KENYA**			
	**Full sample (*n* = 736)**	**Never screened (*n* = 370)**	**Ever screened (*n* = 366)**	***p*-value Never vs. ever screened**^**a**^
Age, average (SD)	37.9 (8.7)	35.9 (8.9)	39.8 (8.0)	< 0.0001^a^
Currently employed: Yes, n (%)	545 (74.6%)	260 (47.7%)	285 (52.3%)	0.021
No	186 (25.4%)	107 (57.5%)	79 (42.5%)	
Marital status: Married, n (%)	490 (67.0%)	240 (49.0%)	250 (51.0%)	0.345
Not married	241 (33.0%)	127 (52.7%)	114 (47.3%)	
Educational attainment: No school or incomplete primary or less, n (%)	45 (6.1%)	28 (62.2%)	17 (37.8%)	0.128
Completed primary	204 (27.7%)	110 (53.9%)	94 (46.1%)	
Completed secondary	211 (28.7%)	105 (49.8%)	106 (50.2%)	
Beyond secondary	276 (37.5%)	127 (46.0%)	149 (54.0%)	
Household income sufficiency in prior year: Sufficient, n (%)	67 (9.2%)	33 (49.3%)	34 (50.8%0	0.706
Just enough	246 (33.9%)	129 (52.4%)	117 (47.6%)	
Insufficient	413 (56.9%)	203 (49.2%)	210 (50.9%)	
Area of residence: Rural (village), n (%)	349 (47.4%)	172 (49.3%)	177 (50.7%)	0.200
Town (trading center)	238 (32.3%)	130 (54.6%)	108 (45.4%)	
Urban (city)	149 (20.2%)	68 (45.6%)	81 (54.4%)	
Social desirability score^b^, mean (SD)	2.1 (1.4)	2.0 (1.5)	2.1 (1.4)	0.22^a^
Number of children, mean (SD)	2.3 (1.1)	2.2 (1.1)	2.3 (1.1)	0.844^a^
	**MALAWI**			
	**Full sample (*n* = 261)**	**Never screened (*n* = 150)**	**Ever screened (*n* = 111)**	***p*-value Never vs. ever screened**^**a**^
Age, average (SD)	39.1 (10.2)	39.4 (10.9)	38.7 (9.3)	0.581^a^
Currently employed: Yes, n (%)	211 (80.8%)	122 (57.8%)	89 (42.2%)	0.815
No	50 (19.2%)	28 (56.0%)	22 (44.0%)	
Marital status: Married, n (%)	193 (74.0%)	105 (54.4%)	88 (45.6%)	0.091
Not married	68 (26.0%)	45 (66.2%)	23 (33.8%)	
Educational attainment: No school or incomplete primary or less, n (%)	181 (69.4%)	115 (63.5%)	66 (36.5%)	0.028
Completed primary	57 (21.8%)	24 (42.1%)	33 (57.9%)	
Completed secondary	15 (5.8%)	7 (46.7%)	8 (53.3%)	
Beyond secondary	8 (3.1%)	4 (50.0%)	4 (50.0%)	
Household income sufficiency in prior year: Sufficient, n (%)	51 (19.5%)	28 (54.9%)	23 (45.1%)	0.916
Just enough	145 (55.6%)	84 (57.9%)	61 (42.1%)	
Insufficient	65 (24.9%)	38 (58.5%)	27 (41.5%)	
Area of residence: Rural (village), n (%)	220 (84.3%)	127 (57.7%)	93 (42.3%)	0.846
Town (trading center)	41 (15.7%)	23 (56.1%)	18 (43.9%)	
Urban (city)	0	n/a	n/a	
Social desirability score^b^, mean (SD)	3.2 (1.0)	3.1 (0.9)	3.3 (1.1)	0.201^a^
Number of children, mean (SD)	3.5 (1.4)	3.5 (1.5)	3.5 (1.4)	0.940^a^

Table excludes missing values; the Kenya dataset is missing employment status for 5 women, missing marital status for 5 women, missing income sufficiency for 10 women, and missing a social desirability score for 4 women; in Malawi, the dataset is missing age for 4 women

^a^*p*-value based on chi-square test for all variables except those indicated with this superscript which indicates the *p*-value is based on a t-test

^b^Can range 0 (low) to 5 (high)

### Perceived susceptibility

On average, women surveyed in Kenya responded correctly to 2.6 questions out of 4 about cervical cancer risk, and women surveyed in Malawi responded correctly to 3.0 questions (Table 3). In addition to an overall higher knowledge score in Malawi, more Malawian than Kenyan women responded correctly to each knowledge question; for example, 80.8% of Malawian respondents correctly identified that HPV can cause cervical cancer, compared to only 64.5% of Kenyan respondents (Table 3).

**Table 3:** Perceived susceptibility factors and association with cervical cancer screening behavior

	**KENYA**			
	**Full sample (*n*= 736)**	**Never screened (*n*= 370)**	**Ever screened (*n* = 366)**	**Adjusted risk ratio**^**a**^ **(95% CI), Ever screened**
HPV can cause cervical cancer, n (%) correct ^b^	475 (64.5%)	229 (61.9%)	246 (67.2%)	1.13 (0.96, 1.32)
HPV can be passed on during sexual intercourse, n (%) correct^b^	428 (58.2%)	194 (52.6%)	234 (63.9%)	1.28** (1.09, 1.50)
Men can get HPV, n (%) correct^b^	328 (44.6%)	155 (41.9%)	173 (47.4%)	1.16* (1.00, 1.33)
A person could have HPV for many years without knowing it, n (%) correct^b^	643 (87.4%)	322 (87.0%)	321 (87.7%)	0.96 (0.76, 1.21)
Overall knowledge score^c^, mean (SD)	2.55 (1.23)	2.43 (1.18)	2.66 (1.23)	1.09* (1.02, 1.16)
	**MALAWI**			
	**Full sample (*n* = 261)**	**Never screened (*n* = 150)**	**Ever screened (*n* = 111)**	**Adjusted risk ratio (95% CI)**^**a**^**, Ever screened**
HPV can cause cervical cancer, n (%) correct^b^	211 (80.8%)	122 (81.3%)	89 (80.2%)	0.96 (0.67, 1.38)
HPV can be passed on during sexual intercourse, n (%) correct^b^	186 (71.3%)	107 (71.3%)	79 (71.2%)	1.05 (0.76, 1.45)
Men can get HPV, n (%) correct^b^	160 (61.3%)	94 (62.7%)	66 (59.5%)	1.09 (0.80, 1.49)
A person could have HPV for many years without knowing it, n (%) correct^b^	235 (90.0%)	135 (90.0%)	100 (90.1%)	1.11 (0.67, 1.82)
Overall knowledge score^c^, mean (SD)	3.03 (1.16)	3.05 (1.17)	3.01 (1.15)	1.02 (0.90, 1.17)

^a^Adjusted risk ratios from Poisson models include: age (continuous), currently employed (yes/no), currently married (yes/no), highest educational attainment (less than primary/primary/secondary/more than secondary), household income sufficiency (sufficient/just enough/not sufficient), area of residence (rural/town/urban), social desirability score (continuous), number of children (continuous)

^b^Correct versus Incorrect or don’t know (excludes prefer not to answer & missing responses)

^c^Sum of above items (1 point per correct), can range 0–4

^*^p < 0.05, ** *p* < 0.01, ***p < 0.001

Overall knowledge about cervical cancer and HPV was significantly associated with screening behavior in Kenya: the adjusted risk ratio of having ever been screened was 1.09 per each additional question answered correctly (95% CI 1.02–1.16, *p* < 0.05) (Table 3). Knowledge of two specific items was associated with screening history: knowing that HPV is sexually transmissible (aRR 1.28, 95% CI 1.09–1.50, *p* < 0.01) and knowing that men can get HPV (aRR 1.16, 95% CI 1.00–1.33, *p* < 0.05) (Table 3). In Malawi, women who had been screened more often answered these correctly than their unscreened peers; however, the association was not statistically significant in the adjusted models (Table 3).

### Perceived severity

In Kenya, 52.7% of women (*n* = 388) knew someone who had cervical cancer and 42.7% of women (*n* = 314) knew someone who had died due to cervical cancer – and these were positively associated with screening behavior, with an adjusted risk ratio of 1.34 for ever versus never screening and knowing someone who had cervical cancer (95% CI 1.15–1.57, *p* < 0.001), and an adjusted risk ratio of 1.3 for ever versus never screening and knowing someone who had died due to cervical cancer (95% CI 1.12–1.51, *p* < 0.001) (Table 4).

**Table 4:** Perceived severity factors and association with cervical cancer screening behavior

	**KENYA**			
	**Full sample (*n* = 736)**	**Never screened (*n* = 370)**	**Ever screened (*n* = 366)**	**Adjusted risk ratio**^**a**^ **(95% CI), Ever screened**
Do you know anyone who has had cervical cancer? Yes n (%)	388 (52.7%)	161 (43.5%)	227 (62.0%)	1.34*** (1.15, 1.57)
Do you know anyone who died due to cervical cancer, Yes n (%)	314 (42.7%)	127 (34.3%)	187 (51.1%)	1.30*** (1.12, 1.51)
	**MALAWI**			
	**Full sample (*n* = 261)**	**Never screened (*n* = 150)**	**Ever screened (*n* = 111)**	**Adjusted risk ratio (95% CI)**^**a**^**, Ever screened**
Do you know anyone who has had cervical cancer?, Yes n (%)	123 (47.3%)	52 (34.7%)	71 (64.6%)	2.03*** (1.49, 2.78)
Do you know anyone who died due to cervical cancer, Yes n (%)	91 (34.9%)	41 (27.3%)	50 (45.1%)	1.46* (1.09, 1.95)

^a^Adjusted risk ratios from Poisson models include: age (continuous), currently employed (yes/no), currently married (yes/no), highest educational attainment (less than primary/primary/secondary/more than secondary), household income sufficiency (sufficient/just enough/not sufficient), area of residence (rural/town/urban), social desirability score (continuous), number of children (continuous)

^*^p < 0.05, ** *p* < 0.01, ***p < 0.001

In Malawi, 47.3% of women (*n* = 123) knew someone who had cervical cancer and 34.9% of women (*n* = 91) knew someone who had died due to cervical cancer, and these were strongly and significantly associated with screening behavior; the adjusted risk ratio of ever versus never screening and knowing someone who had cervical cancer was 2.03 (95% CI 1.49–2.78, *p* < 0.001) and knowing someone who died due to cervical cancer was 1.46 (95% CI 1.09–1.95, *p* < 0.05) (Table 4).

### Perceived barriers

In both countries, access to preventive health care (proxied by access to routine vaccination services and HPV vaccination services for the respondent’s child/children) was good: the average rating for access difficulties was approximately 1, on a scale of 1–4 where 1 represents “not at all difficult” (Table 5). In Malawi (but not in Kenya), those with easier reported access to routine vaccination services for their child/children also had a higher adjusted risk ratio of having been screened for cervical cancer (aRR 1.34, 95% CI 1.16–1.55, *p* < 0.001) (Table 5).

**Table 5:** Perceived barrier and benefit factors and association with cervical cancer screening behavior

	**KENYA**			
	**Full sample (*n* = 736)**	**Never screened (*n* = 370)**	**Ever screened (*n* = 366)**	**Adjusted risk ratio**^**a**^ **(95% CI), Ever screened**
Access score for routine vaccines, mean (SD)^b^	1.15 (0.53)	1.13 (0.49)	1.17 (0.57)	1.06 (0.94, 1.19)
Access score for HPV vaccine, mean (SD)^c^	1.39 (0.84)	1.40 (0.86)	1.38 (0.82)	0.99 (0.91, 1.07)
Trust in information about HPV vaccination from Ministry of Health/government, A lot or some n (%)^d^	699 (95.0%)	352 (95.1%)	347 (94.8%)	1.01 (0.72, 1.42)
Trust in information about HPV vaccination from doctors and nurses, A lot or some n (%)^d^	705 (95.8%)	352 (95.1%)	353 (96.5%)	1.16 (0.76, 1.78)
	**MALAWI**			
	**Full sample (*n* = 261)**	**Never screened (*n* = 150)**	**Ever screened (*n* = 111)**	**Adjusted risk ratio (95% CI)**^**a**^**, Ever screened**
Access score for routine vaccines, mean (SD)^b^	1.15 (0.50)	1.09 (0.37)	1.24 (0.64)	1.34*** (1.16, 1.55)
Access score for HPV vaccine, mean (SD)^c^	1.29 (0.72)	1.32 (0.80)	1.24 (0.58)	0.88 (0.70, 1.10)
Trust in information about HPV vaccination from Ministry of Health/government, A lot or some n (%)^d^	247 (94.6%)	142 (94.7%)	105 (94.6%)	1.06 (0.57, 1.95)
Trust in information about HPV vaccination from doctors and nurses, A lot or some n (%)^d^	252 (96.6%)	146 (97.3%)	106 (95.5%)	0.78 (0.42, 1.45)

^a^Adjusted risk ratios from Poisson models include: age (continuous), currently employed (yes/no), currently married (yes/no), highest educational attainment (less than primary/primary/secondary/more than secondary), household income sufficiency (sufficient/just enough/not sufficient), area of residence (rural/town/urban), social desirability score (continuous), number of children (continuous)

^b^Score can range 1–4 and represents the rating of access difficulty for routine childhood vaccines (1 represents “not at all difficult” and 4 represents “very difficult”)

^c^Score can range 1–4 and represents the average rating of access difficulty (anticipated or experienced) for first and second doses of the HPV vaccine (1 represents “not at all difficult” and 4 represents “very difficult”)

^d^A lot or some, versus not much or not at all or prefer not to answer

^*^p < 0.05, **p < 0.01, ***p < 0.001

### Perceived benefits

Nearly all respondents—approximately 95%—in both countries trusted health information about cervical cancer prevention (the HPV vaccine) from the Ministry of Health/government, and from doctors and nurses; and this was not significantly associated with screening behavior in adjusted models in either country (Table 5).

### Self-efficacy

Cervical cancer screening was more common among respondents who had received the COVID-19 vaccine (Kenya aRR 1.45 [95% CI 1.12–1.87, *p* < 0.001], Malawi aRR 1.371 [95% CI 1.03–1.82, *p* < 0.05]) (Table 6). Overall, nearly twice as many Kenyan respondents had received any doses of the COVID-19 vaccine than Malawian respondents (83.1% versus 42.5% of the samples, respectively) (Table 6).

**Table 6:** Self-efficacy and cues to action factors and association with cervical cancer screening behavior

	**KENYA**			
	**Full sample (*n* = 736)**	**Never screened (*n*** **= 370)**	**Ever screened (*n* = 366)**	**Adjusted risk ratio**^**a**^ **(95% CI), Ever screened**
Received any doses of the COVID-19 vaccine	610 (83.1%)	288 (78.3%)	322 (88.0%)	1.45** (1.12, 1.87)
Ever talked with a health worker about cervical cancer or the HPV vaccine	378 (51.4%)	131 (35.4%)	247 (67.5%)	1.88*** (1.59, 2.24)
Ever talked with other parents about cervical cancer or the HPV vaccine	410 (55.7%)	178 (48.1%)	232 (63.4%)	1.32** (1.12, 1.55)
	**MALAWI**			
	**Full sample (*n* = 261)**	**Never screened (*n* = 150)**	**Ever screened (*n* = 111)**	**Adjusted risk ratio (95% CI)**^**a**^**, Ever screened**
Received any doses of the COVID-19 vaccine	111 (42.5%)	56 (37.3%)	55 (49.6%)	1.37* (1.03, 1.82)
Ever talked with a health worker about cervical cancer or the HPV vaccine	120 (46.0%)	53 (35.3%)	67 (60.4%)	1.89*** (1.41, 2.54)
Ever talked with other parents about cervical cancer or the HPV vaccine	128 (49.0%)	60 (40.0%)	68 (61.3%)	1.71*** (1.27, 2.29)

^a^Adjusted risk ratios from Poisson models include: age (continuous), currently employed (yes/no), currently married (yes/no), highest educational attainment (less than primary/primary/secondary/more than secondary), household income sufficiency (sufficient/just enough/not sufficient), area of residence (rural/town/urban), social desirability score (continuous), number of children (continuous)

^*^p < 0.05, ** *p* < 0.01, ***p < 0.001

### Cues to action

Although only approximately half of Kenyan and Malawian women had ever spoken to a health worker or to other parents about cervical cancer or HPV vaccination, those who had were significantly more likely to have ever been screened (Table 6). In Kenya, the adjusted risk ratio for having been screened for those who had talked with a health worker about cervical cancer prevention was 1.88 (95% CI 1.59–2.24, *p* < 0.001) and in Malawi the adjusted risk ratio was 1.89 (95% CI 1.41–2.54, *p* < 0.001) (Table 6). Likewise, Kenyan respondents who had spoken with other parents about cervical cancer prevention had an adjusted risk ratio of screening of 1.32 (95% CI 1.12–1.55, *p* < 0.01) and in Malawi the adjusted risk ratio was 1.71 (95% CI 1.27–2.29, *p* < 0.001) (Table 6).

## Discussion

In this study, just under half of surveyed women in Kenya and Malawi reported ever having been screened for cervical cancer. In both countries, cues to action (speaking with other people about cervical cancer and cervical cancer prevention) and perceived severity (knowing someone affected by cervical cancer) were strongly associated with cervical cancer screening history. Other factors differed by country: in Kenya, perceived susceptibility was an important determinant as were woman’s age and employment, while in Malawi, barriers to accessing preventive care were significantly associated with screening as was higher educational attainment. Importantly, due to differences in sample selection, we did not pool the data and statistically test whether these relationships are significantly different in the two contexts.

Women in this study more commonly reported having ever been screened for cervical cancer than has been reported in previous studies from these countries: 49.7% of Kenyan respondents and 42.5% of Malawian respondents; while recent nationally-representative household surveys had found ever-screening rates of 17% among all surveyed women (aged 15 years and older) in both countries [29, 30]. We have several hypotheses for why our study found a higher screening rate than these previous studies. First, these previous studies were nationally representative but ours were not, and differences in the samples may lead to differences in cervical cancer screening behavior. For example, in Malawi the household survey was conducted in only three districts and we did not measure any area-level factors (e.g., availability of screening services or a recent screening promotion campaign) which could have boosted screening rates locally; and in Kenya, phone survey respondents may differ from the general population in ways that matter for screening uptake. In addition, nearly all respondents in our study were between the ages of 35 and 50 years (due to the parent study’s eligibility criteria); as this is the priority group for cervical cancer screening [44], this sample may have over-representation of the ages most commonly screened. Screening has been found to be more prevalent for women of these ages than their younger and older peers in both countries. There also may have been social desirability or other reporting bias in our study, if respondents were inclined to say they had been screened when asked. In an attempt to address this, our adjusted models do include a social desirability score, but it is impossible to rule out this type of bias.

We found few sociodemographic differences between women who had ever been screened for cervical cancer and those who had not. In contrast to previous studies from Kenya [31, 39, 45–48] and Malawi [33, 49–51], and elsewhere in the region [16, 17, 19, 52, 53], we did not find that women of lower reported household wealth were less commonly screened than their wealthier peers. This may be attributable to scale-up of national screening programs, if lower-income populations are increasingly reached through these efforts. It may also reflect the non-representative samples in this analysis: in Kenya, we conducted a mobile phone survey which may disproportionately capture wealthier respondents; and in Malawi, we surveyed only in selected geographic areas so may have had limited variation in some socioeconomic and demographic characteristics.

We found relatively high knowledge about cervical cancer risk in Malawi (in contrast to several previous studies [50, 54–56]), and lower knowledge in Kenya (in agreement with other studies [40, 57]). The association seen here between perceived susceptibility and cervical cancer screening behavior has been reported in other studies from Kenya [35, 57–59], suggesting that an educational or awareness-raising intervention that focuses on risk factors for cervical cancer may be an effective approach for increasing screening uptake in this context. In addition, many survey respondents had first-hand knowledge of someone who had experienced cervical cancer, and 34.9% of Malawian women and 42.7% of Kenyan women knew someone who had died due to cervical cancer. This perceived risk was also strongly associated with women’s own cervical cancer screening behavior, as has been identified in previous studies from these countries [34, 58, 60]. Therefore, an intervention that leverages peers who were affected by cervical cancer may be particularly high-impact for women in Kenya and Malawi.

Other studies have found that women face substantial barriers to cervical cancer screening services [18–20, 61], including research specifically from Kenya [39, 40, 58, 62] and Malawi [63, 64]. Our research found an association between cervical cancer screening and access to other preventive health services only in Malawi. We used an imperfect proxy for access (access to vaccines for the child/children), particularly since access is a multifaceted construct and there may be unique aspects to cervical cancer screening access – including availability of supplies, equipment and trained providers (which have been identified as barriers in these countries [38, 63, 65, 66]) – which may not affect perceived or reported access to other health services. More dedicated research is needed about the specific aspects of access to cervical cancer screening that may pose barriers for women in these contexts.

Among respondents in Kenya, self-efficacy to engage in preventive health care, measured here as having received a COVID-19 vaccination, was strongly associated with women’s cervical cancer screening history. This relationship was present but not statistically significant among women in Malawi. It is worth noting that COVID-19 vaccination behavior is highly complex and may not perfectly reflect self-efficacy to engage in cervical cancer screening. Prior studies have likewise found that other health behaviors like condom use are correlated with Kenyan women’s cervical cancer screening behavior [40, 67], and that women who had been screened previously were more likely to be screened again compared to never-screeners [32, 68]. We also found a very strong association between screening and speaking with other people, including with health care workers, about cervical cancer and cervical cancer prevention (also identified in previous studies from Malawi specifically [51, 54, 55, 63]). It is possible that these conversations are concurrent with the screening service, and this survey did not ask about the sequence of these events so we can only assess association (not causality). However, taken together, these results suggest that interventions may seek to leverage health workers as trusted messengers to promote cervical cancer screening during other health activities.

Several limitations to this study should be noted. First, the modality and sample for data collection differed in the two countries. In Kenya we conducted a national survey via mobile phone due to very high mobile phone penetration (97% of Kenyans own a mobile phone [69]), and in Malawi we collected data via a household survey in three districts, since mobile phone access is variable. For this reason, we conducted separate analyses in the two countries, and generalizability of the findings may be limited. There are limitations related to each data collection approach: for example, the sampling frame for mobile phone surveys may over-represent people of higher socioeconomic status, greater educational attainment, and in urban areas. The results should be interpreted in light of the unique biases inherent to each sampling approach. Second, there are limitations to how we operationalized constructs of the Health Belief Model (many of which have not been validated for this topic in this setting). For example, as noted above, the construct of “barriers” may have been imperfectly represented by access to other preventive health care services (vaccinations), and the construct of “self-efficacy” was represented here by vaccination behavior which may be very different in nature from engaging in screening. Related, there may be other factors associated with cervical cancer screening that were not explored in this analysis – e.g., spousal or social support – and future studies should seek to include these.

## Conclusions

This paper generates new insights about factors that may contribute to cervical cancer screening in two high-burden contexts, and suggests potential areas of intervention such as leveraging experiences of women with a history of cervical cancer, and promoting health worker recommendation of screening to eligible women. The findings also suggest the need for tailored interventions in different settings; for example, integrating cervical cancer screening with other preventive health behaviors, working to increase knowledge about cervical cancer and its prevention, and easing access to screening services.

## Supplementary Information

Supplementary Material 1.

## Data Availability

The datasets generated and analyzed during the current study will not be publicly available because the research participants did not consent to this, but a deidentified dataset will be available from the corresponding author on reasonable request.

## Abbreviations

LMICs: Low- and Middle-Income Countries
HPV: Human Papillomavirus
WHO: World Health Organization
HBM: Health Belief Model
SDRS-5: Socially Desirable Response Set (five-item version)
aRR: Adjusted Risk Ratio
CI: Confidence Interval
SD: Standard Deviation
