# Supplementary material for: Factors associated with cervical cancer screening: results from cross-sectional surveys in Kenya and Malawi
Source: BMC Public Health. 2025 May 27;25:1956. doi: 10.1186/s12889-025-23143-y (PMC12107903; doi:10.1186/s12889-025-23143-y)
Supplement: Supplementary file 1 — Supplementary Material 1. [file 12889_2025_23143_MOESM1_ESM.docx]

**Appendix 1**: Questions from survey tool used in this analysis

| What is your current age? | ___________ (complete years)  ☐ Don’t know/Prefer not to answer |
| --- | --- |
| What is the highest level of school you completed? (select one) | ☐ Primary  ☐ Secondary  ☐ Higher  ☐ No school/Did not complete primary  ☐ Don’t know/Prefer not to answer |
| What is your current employment status? (select one) | □ Employed full-time  □ Employed part-time  □ Casual laborer  □ Self-employed  □ Not employed but looking for work  □ Not employed and not looking for work   Don’t know/Prefer not to answer |
| What is your current marital status? (select one) | □ Single  □ Cohabiting/Partnered  □ Married  □ Widowed  □ Divorced/Separated   Don’t know/Prefer not to answer |
| Over the past 12 months, how would you describe your household income? |  Allowed me to build my savings   Allowed me to save just a little   Only just met my expenses   Was not sufficient, so needed to use savings to meet expenses   Was really not sufficient, so needed to borrow to meet expenses   Don’t know/Prefer not to answer |
| How would you describe the area where you live at present? Is it a… |  City (urban)   Trading Center (town)   Village (rural) |
| For how many children do you participate in decision-making, or make decisions, about vaccinations? | ___ (number of children) |
| Overall how difficult do you feel it is/was to get routine childhood vaccines (under 5 vaccines – like polio, measles, etc.)? | ☐ Not at all difficult  ☐ A little difficult  ☐ Somewhat difficult  ☐ Very difficult   Don’t know/Prefer not to answer |
| For those with daughter(s) who have 0 HPV vaccine doses: Overall how difficult do you feel it would be to get an HPV vaccine for your daughter? |  |
| For those with daughter(s) who have 1+ HPV vaccine doses: Overall how difficult do you feel it was to get the first dose of HPV vaccine for your daughter? |  |
| For those with daughter(s) who have 2+ HPV vaccine doses Overall how difficult do you feel it was to get a second dose of the HPV vaccine for your daughter? |  |
| Have you ever talked about cervical cancer or HPV vaccine with other parents? | ☐ Yes  ☐ No |
| Have you ever talked about cervical cancer or HPV vaccine with a doctor / nurse / other health care worker? |  |
| In general, how much do you trust HPV vaccine information & advice from the government (Ministry of Health)? |  A lot   Some   Not much   Not at all   Don’t know/Prefer not to answer |
| In general, how much do you trust HPV vaccine information & advice from doctors and nurses? |  |
| I am going to ask a few questions about HPV and cervical cancer. You should answer each one True or False, or I don’t know | |
| HPV can cause cervical cancer |  True   False   Don’t know/Prefer not to answer |
| HPV can be passed on during sexual intercourse |  |
| Men can get HPV |  |
| A person could have HPV for many years without knowing it |  |
| Do you know anyone who has had cervical cancer? (can check more than 1) |  Yes, I know someone who had cervical cancer and is still alive   Yes, I know someone who died due to cervical cancer   No   Don’t know/Prefer not to answer |
| (For female respondents only) Have you ever been screened/tested for cervical cancer? |  Yes   No   Unsure   Don’t know/Prefer not to answer |
| I am going to read a few statements about your relationships with others. For each, please indicate how much it is true or false for you | |
| I am always courteous even to people who are disagreeable |  Definitely true   Mostly true   Mostly false   Definitely false   Don’t know/Prefer not to answer |
| There have been occasions when I took advantage of someone. |  |
| I sometimes try to “get even” rather than “forgive and forget.” |  |
| I sometimes feel resentful when I don’t get my way. |  |
| No matter who I am talking to, I’m always a good listener. |  |
| Have you received a vaccine against COVID-19? | □ Yes, 1 dose and I am fully vaccinated (1-dose vaccine type)  □ Yes, 2 doses and I am fully vaccinated (2-dose vaccine type)  □ Yes, 1 dose and I am partially vaccinated (2-dose vaccine type)  □ No, 0 doses   Don’t know/Prefer not to answer |
